# Hepatic Organoid-Based High-Content Imaging Boosts Evaluation of Stereoisomerism-Dependent Hepatotoxicity of Stilbenes in Herbal Medicines

**DOI:** 10.3389/fphar.2022.862830

**Published:** 2022-05-17

**Authors:** Juan Liu, Tingting Li, Ruihong Li, Jie Wang, Pengyan Li, Ming Niu, Le Zhang, Chunyu Li, Tao Wang, Xiaohe Xiao, Jia-bo Wang, Yunfang Wang

**Affiliations:** ^1^ Hepato-Pancreato-Biliary Center, Beijing Tsinghua Changgung Hospital, Tsinghua University, Beijing, China; ^2^ Integrative Medical Center, The Fifth Medical Centre, Chinese PLA General Hospital, Beijing, China; ^3^ Stem Cells and Tissue Engineering Lab, Institute of Health Service and Transfusion Medicine, Beijing, China; ^4^ Nephrology Combined with Traditional Chinese and Western Medicine, The First Hospital of Hebei Medical University, Shijiazhuang, China; ^5^ School of Traditional Chinese Medicine, Capital Medical University, Beijing, China

**Keywords:** hepatotoxicity, organoids, high-content imaging, stereoisomer, *Polygonum multiflorum*

## Abstract

The complexity of chemical components of herbal medicines often causes great barriers to toxicity research. In our previous study, we have found the critical divergent hepatotoxic potential of a pair of stilbene isomers in a famous traditional Chinese herb, *Polygonum multiflorum* (Heshouwu in Chinese). However, the high-throughput *in vitro* evaluation for such stereoisomerism-dependent hepatotoxicity is a critical challenge. In this study, we used a hepatic organoids–based *in vitro* hepatotoxic evaluation system in conjunction with using high content imaging to differentiate *in vivo* organ hepatotoxicity of the 2,3,5,4′-tetrahydroxy-*trans-*stilbene-2-O-β-glucoside (*trans-SG*) and its *cis-*isomer (*cis-SG*). By using such an organoid platform, we successfully differentiated the two stereoisomers’ hepatotoxic potentials, which were in accordance with their differences in rodents and humans. The lesion mechanism of the toxic isomer (*cis-SG*) was further found as the mitochondrial injury by high-content imaging, and its hepatotoxicity could be dose-dependently inhibited by the mitochondrial protective agent. These results demonstrated the utility of the organoids-based high-content imaging approach in evaluating and predicting organ toxicity of natural products in a low-cost and high-throughput way. It also suggested the rationale to use long-term cultured organoids as an alternative toxicology platform to identify early and cautiously the hepatotoxic new drug candidates in the preclinical phase.

## Introduction

There is a safety problem in the clinical use of Chinese herbal medicine because of its constituent elements being complex. Especially, hepatotoxicity associated with herbal or botanical use is increasingly being recognized, as the use of these medicines has become widespread in many countries (Teschke et al., 2015; Liu et al., 2019; Björnsson and Björnsson 2022). Hundreds of Chinese medicines or their extracts have been reported to possess potential hepatotoxicity, which results in severe clinical adverse events, such as liver fibrosis, hepatitis, liver failure, and even death. Suspected cases of Chinese medicine–induced liver injury have been highlighted in many publications on case reports and case series (Hebels et al., 2014; Zhang et al., 2018).

**FIGURE 1:**
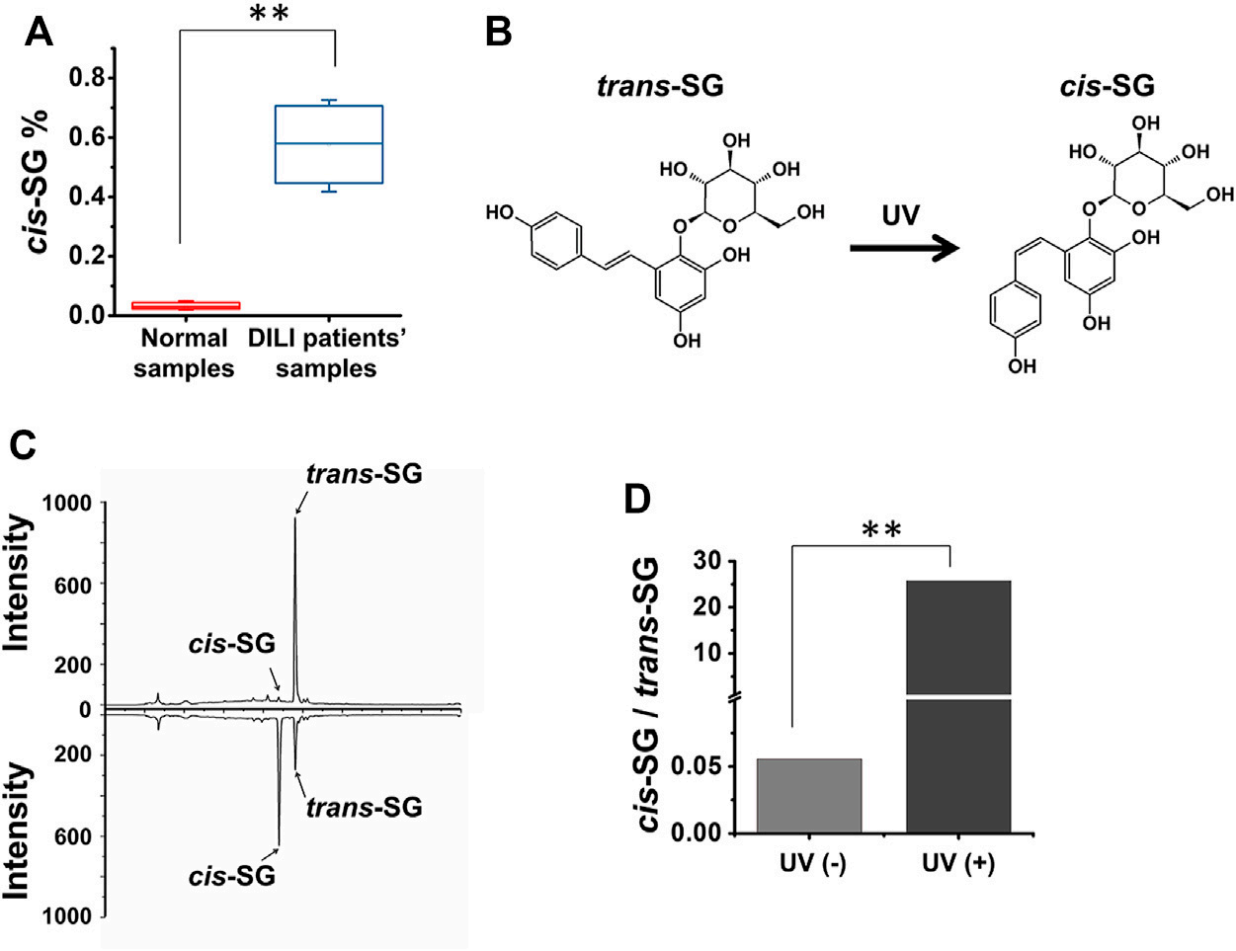
*Cis-SG* in *Polygonum multiflorum*–induced DILI patients’ samples and its photoisomerization. **(A)** The content of *cis-SG* in normal *Polygonum multiflorum* samples and in samples from DILI patients. **(B)** The chemical structure of *trans-SG* and *cis-SG*. **(C)** The HPLC results of *trans-SG* and *cis-SG* without or with UV radiation. **(D)** The proportion of *cis-SG* to *trans-SG* without and with UV treatment. Values are expressed as mean ± SD. **p < 0.01.

*Polygonum multiflorum* (Heshouwu in Chinese), a widely used traditional Chinese medicine, has the functions of detoxification, carbuncle elimination, bowel relaxation, and malaria prevention and has also been recommended for the nourishment of the liver for many years. Recently, raw *P. multiflorum* has become popular in Europe and the United States as herbal and dietary supplements (Liu et al., 2018). Nevertheless, a series of reports of adverse hepatotoxic effects induced by raw *P. multiflorum*–containing drugs (Shou Wu Pian, Shen Min, etc.) have been occasionally reported in recent years, arousing great public concerns, and have also been summarized and recorded in the LiverTox^®^ database (He et al., 2019). The hepatotoxicities induced by *P. multiflorum* are difficult to be defined due to its complex components and multiple functions (Li et al., 2017; Liu et al., 2018). Our previous work has shown that cotreatment with a nontoxic dose of lipopolysaccharide (LPS) and a therapeutic dose of *P. multiflorum* can induce liver injury in rats, whereas the solo treatment cannot induce observable injury (Dong et al., 2015; Li et al., 2017; Li et al., 2019; Zhang et al., 2020). The bioactive components of *P. multiflorum* include tetrahydroxystilbene glucoside, anthraquinone, phospholipids, and tannins. The 2,3,5,4′-tetrahydroxy-*trans*-stilbene-2-O-β-glucoside (*trans-SG*) as the major constituent has been concerning as the major hepatotoxic component in *P. multiflorum* (Wang et al., 2015). However, limited evidence exists to support this conclusion. The *trans-*isomer of stilbenes, such as *trans-SG*, can be transformed by ultraviolet (UV) light or sunlight into its cis-isomer (*cis-SG*). Stereoisomers account for quite a large percentage in medicines and have extremely similar structures but great differences in pharmacodynamics and pharmacokinetics, as well as in drug-induced toxicity. Interestingly, we collected *P. multiflorum* samples from patients who had experienced liver injury and found higher *cis-SG* content (Figure 1A). The opportunities for exposure to UV radiation in the course of sunlight drying, preparation, and preservation of the herb are known to be abundant. We still do not know the possible association of UV-induced *cis-trans* isomerization of *trans-SG* with the idiosyncratic hepatotoxicity of *P. multiflorum*.

Predicting and studying the hepatotoxicity induced by drugs remain challenging for human health and pharmaceutical companies. As hepatotoxicity is one of the major reasons for developmental failures and withdrawal of new drugs, the development of evaluating models is needed to assess drugs and study the possible mechanisms of drug-induced liver injury (DILI) (Njoku 2014; Shehu et al., 2017). Nowadays, the development of innovative technologies brings opportunities for DILI forecasting, making it high throughput and highly sensitive and of low cost (Gomez-Lechon and Tolosa 2016; Funk and Roth 2017; Kuna et al., 2018). By using the techniques of molecular imaging, high-content screening, and cellular phenotype on suitable human hepatic cell models, most of the hepatotoxic-related molecular pathways can be determined, and they might be extended to the reactions at the tissue and organ levels to predict drug toxicity from molecular initiation to harmful outcome paths (Xu et al., 2008; Garside et al., 2014; Wink et al., 2018). In our previous work, we developed a simple but robust human-specific 3D HepaRG cell culture platform based on native liver extracellular matrix with multiparametric readouts to analyze the hepatotoxicity and possible mechanisms induced by drugs (Juan et al., 2018). The 3D cultured model, which has tight intracellular junctions, high-level expressions of metabolizing enzymes, and the ability of long-term culturing, has shown promising advantages in the evaluation of drug hepatotoxicity with simulation to *in vivo* organ functions. However, data about the utility of the 3D model in evaluating hepatotoxic differences between critically similar chemical structures have been sparse. Therefore, in this study, we tried to assess the possible hepatotoxic differences between a pair of stereoisomers, *trans-* and *cis-SG*, and to further elucidate the lesion mechanisms simultaneously.

## Materials and Methods

### Compounds and Reagents

2,3,5,4′-tetrahydroxy-*trans-*stilbene-2-O-β-glucoside (*trans-SG*) and its *cis-*isomer (*cis-SG*), as well as Schisandrin B (Sch B), were provided by the Chengdu Chroma Biotechnology Co., Ltd. The reference solutions (0.2 mg/ml) of *cis-SG* and *trans-SG* were prepared separately by methanol and stored in brown bottles. Lipopolysaccharide (LPS) was purchased from Sigma-Aldrich (Lot#113M4068V).

### Sample Collection and Preparation

The *P. multiflorum* samples collected from patients were prepared through extraction for eight times using 50% ethanol-water solution *via* the ultrasound extraction method. *Trans-SG* was weighed precisely, dissolved in methanol to a concentration of 5 mg/ml, placed in a plain dish, and irradiated with UV light at 365 nm for 60 min. After volatilizing methanol in the fume cupboard, the samples were sealed and preserved in light-proof bags. The concentrations of *cis-SG* and *trans-SG* were determined by high-performance liquid chromatography.

### Animal Model and *In Vivo* Hepatotoxicity Evaluation

Male Sprague Dawley (SD) rats weighing 200 ± 10 g were obtained from the Laboratory Animal Center of the Academy of Military Medical Sciences. They were housed in the Laboratory Animal Center of Military Medical Sciences and maintained under a 12-h light/dark cycle in a controlled temperature (25 ± 2°C) and humidity (50–60%) environment for a period of 1 week before evaluation. The animals were allowed access to food and water ad libitum.

The differential hepatotoxicity potential of these two stereoisomers was evaluated by a previously established rat model (Li et al., 2017), which used a nontoxic dosage of LPS pretreatment to induce minimal inflammatory stress. In a typical procedure, the animals were intragastrically administered with *cis-SG* or *trans-SG* or an equivalent volume of normal saline, separately, followed by a tail vein injection of LPS (2.8 mg/kg, Sigma-Aldrich) or normal saline 3 h later. At 7 h after the injection of LPS, the blood and liver samples were collected. The rat liver was fixed in 10% neutral buffered formalin for at least 72 h before being processed for histologic analysis. Paraffin-embedded sections were cut to 4-mm-thick pieces and stained with hematoxylin and eosin for microscopic examination. AST and ALT were also detected.

### Cells and Organoids Culturing

Human hepatoma cell lines and HepaRG cells were obtained from the Chinese Academy of Food and Drug Administration and cultured in Williams’ Media E (Gibco, United States) supplemented with 10% fetal bovine serum (FBS, Gibco, Canada), 2 mM GlutaMAX (Gibco, United States), 100 units/ml penicillin, and 100 μg/ml streptomycin. The medium was renewed every 2 days. The cells were maintained at 37°C with 5% CO_2_ in a cell incubator with a water reservoir.

To prepare the hepatic organoids, the HepaRG cells (400 cells per well) with liver extracellular matrix were seeded into round-bottomed 96-well plates with an ultra-low attachment surface (Costa, Corning) and were incubated at 37°C with 5% CO_2_. The culture medium was partially replaced by a fresh medium every 3 days.

### *In Vitro* Evaluation of Hepatotoxicity

Cell viability was detected by *trans-SG* and *cis-SG*. For the 2D cultured cell model, 100 μL of culture medium containing cells at a density of 8,000 cells/well was plated in 96-well plates, and after incubation of 24 h, the cells were treated with the drugs for 24 h. As for the 3D cultured HepaRG organoids model, the HepaRG cells were cultured under 3D culturing conditions for 14 days to form the organoids, and the organoids were then treated with testing drugs for 24 h once (as a single treatment) or for three repeated times of exposure at day 0, day 3, and day 5 and tested at day 7 (as repeated treatment). Cell viability was assessed using Alamar blue (Invitrogen) according to the manufacturer’s instructions. A microscope was used to observe the change of organoids.

The levels of lactic dehydrogenase (LDH), aspartate transaminase (AST), and alanine aminotransferase (ALT) in the supernatant gathered after treatment with drugs at different concentrations were tested using a reagent kit (Nanjing Jiancheng Bioengineering Institute, Nanjing, China) according to the manufacturer’s instructions.

### High-Content Imaging and Analysis

High-content imaging and analysis of organoids were performed using the Operetta High-Content Imaging System (PerkinElmer). The organoids were treated with *trans-SG* and *cis-SG* for 24 h and washed three times with PBS, and the corresponding probes were added to find the potential lesion mechanisms. All the fluorescence probes used in this study were purchased from Thermofisher. Live and dead cells were detected by calcein AM and EthD-1 probe (LIVE/DEAD™, L3224, 8 μM). Oxidative stress was detected by CellROX and mBcl probe (CellROX™ Deep Red Reagent, C10422, 5 Μm; Monochlorobimane, M1381MP, 100 μM). Mitochondria damage was detected by the MitoTracker probe (MitoTracker^®^ Deep Red FM, M22426, 200 nM). Steatosis was detected by a Nile Red probe (Nile Red, N1142, 1 μM). Cholestasis was detected by the CMFDA probe (5-chloromethylfluorescein diacetate, C7025, 25 μM). All the probes were incubated for 30 min, the cells were washed three times with PBS, and pictures were taken and analyzed in 3D space.

### Mitochondrial Protection Compound Screening by Network Pharmacology

The chemical compounds in traditional Chinese medicine and their targets were acquired from the TCM-SP and the TCM Database@Taiwan. We then used these keywords, namely, mitochondrial depolarization, depolarized mitochondria, damaged mitochondria, mitochondrial disorders, mitochondrial dysfunction, mitochondrial impairment, and mitochondrial damage, to retrieve the corresponding target information and establish the mitochondrial lesion target database for the subsequent analysis. Based on the Database of Interacting Proteins (DIP), the interacting network between drug compounds and mitochondrial lesion–related targets was established. The network characteristic parameters of the key node proteins, such as degree (D), betweenness centrality (BC), and closeness centrality (CC), were calculated. For the key node proteins with a D value greater than the median of the D value of all nodes, the key drug targets were screened out from the node proteins whose BC and CC values were greater than the median of the two parameters of all nodes. The screen parameters were set as CC ≥ 0.183, D ≥ 2, and BC ≥ 0.000906 and a total of 19 targets were selected according to the network analysis results. Based on the established compound–target database, the candidate mitochondria protective agents were predicted and selected by using a hypergeometric distribution model to enrich the compound–target interaction network involving the 19 targets (*p <* 0.01).

### Clinical Efficacy Evaluation of Selected Mitochondrial Protection Compounds

The DILI cases caused by PM which were then treated with preparations containing Sch B were collected from the Fifth Medical Centre, Chinese PLA General Hospital (inpatient records between 2007 and 2016).

### qRT-PCR Analysis

Total RNA was isolated using the RNeasy Mini Kit (Qiagen), after which cDNA was synthesized with reverse transcriptase (ReverTra Ace^®^ qPCR RT Master Mix, Toyobo) according to the manufacturer’s instructions. The qRT-PCR was performed with SYBR Green Master Mix (TOYOBO) on a Bio-Rad iQ5 real-time PCR detection system. Data were collected using the Bio-Rad CFX Manager software, and the expression of genes within a sample is normalized to the GAPDH expression by the 2^−ΔΔCt^ method. The primers used in this study are listed in Table 1.

**TABLE 1:** The primers used in this study.

Gene	Forward primer	Reverse primer
GAPDH	CAT​GAG​AAG​TAT​GAC​AAC​AGC​CT	AGT​CCT​TCC​ACG​ATA​CCA​AAG​T
BAX	CCC​GAG​AGG​TCT​TTT​TCC​GAG	CCA​GCC​CAT​GAT​GGT​TCT​GAT
BCL2	GGT​GGG​GTC​ATG​TGT​GTG​G	CGG​TTC​AGG​TAC​TCA​GTC​ATC​C
Caspase3	CAT​GGA​AGC​GAA​TCA​ATG​GAC​T	CTG​TAC​CAG​ACC​GAG​ATG​TCA
PCNA	CCT​GCT​GGG​ATA​TTA​GCT​CCA	CAG​CGG​TAG​GTG​TCG​AAG​C

### Flow Cytometry

The 3D cultured organoids were isolated as a single cell separately by trypsinization, washed three times with PBS, and made into a cell suspension. The MitoProbe™ JC-1 Assay Kit (Invitrogen, M34152) was used to detect mitochondrial membrane potential. The cell suspension was incubated for 20 min with 10 ug/ml JC-1 at 37°C in the dark. The cells were incubated for 15 min with the FITC-Annexin V/Dead Cell Apoptosis Kit (ThermoFisher company, V13242) in the dark at room temperature for early apoptosis and immediately analyzed by flow cytometry according to the instructions. The TUNEL assay was used to detect late apoptosis, and the cells were incubated only with PI for the cell cycle analysis according to the instructions.

### Statistical Analysis

All statistical data are expressed as means and SDs. Unpaired two-tailed Student’s t test was used to compare differences between groups. Differences were considered statistically significant if the *p*-value was smaller than 0.05. Significance was indicated as *p < 0.05, **p < 0.01, and ***p < 0.001. Experiments were repeated at least three times.

## Results

### The Conversion of *Trans*-*SG* to *Cis-SG* Stereoisomer

We collected the rest *P. multiflorum* from the patients, who had been diagnosed as PM—induced liver injury, and tested the content of *cis-SG* in these samples and normal samples using high-performance liquid chromatography (HPLC). As shown in Figure 1A, there was a significant difference in the *cis-SG* content in the DILI patients’ samples which was much higher than in the normal samples. To our knowledge, the *trans-SG* to *cis-SG* conversion occurred under UV light conditions (Figure 1B). In HPLC fingerprinting, we observed that the intensity of *cis-SG* and *trans-SG* was changed after UV light radiation (Figure 1C). Most of the *trans-SG* converted to *cis-SG* with UV light treatment (Figure 1D), indicating that the illumination is important for the storage of *P. multiflorum*.

### The *Cis-SG* Showed Higher Hepatotoxicity in LPS-Treated Rat Model Than *Trans-SG*

**FIGURE 2:**
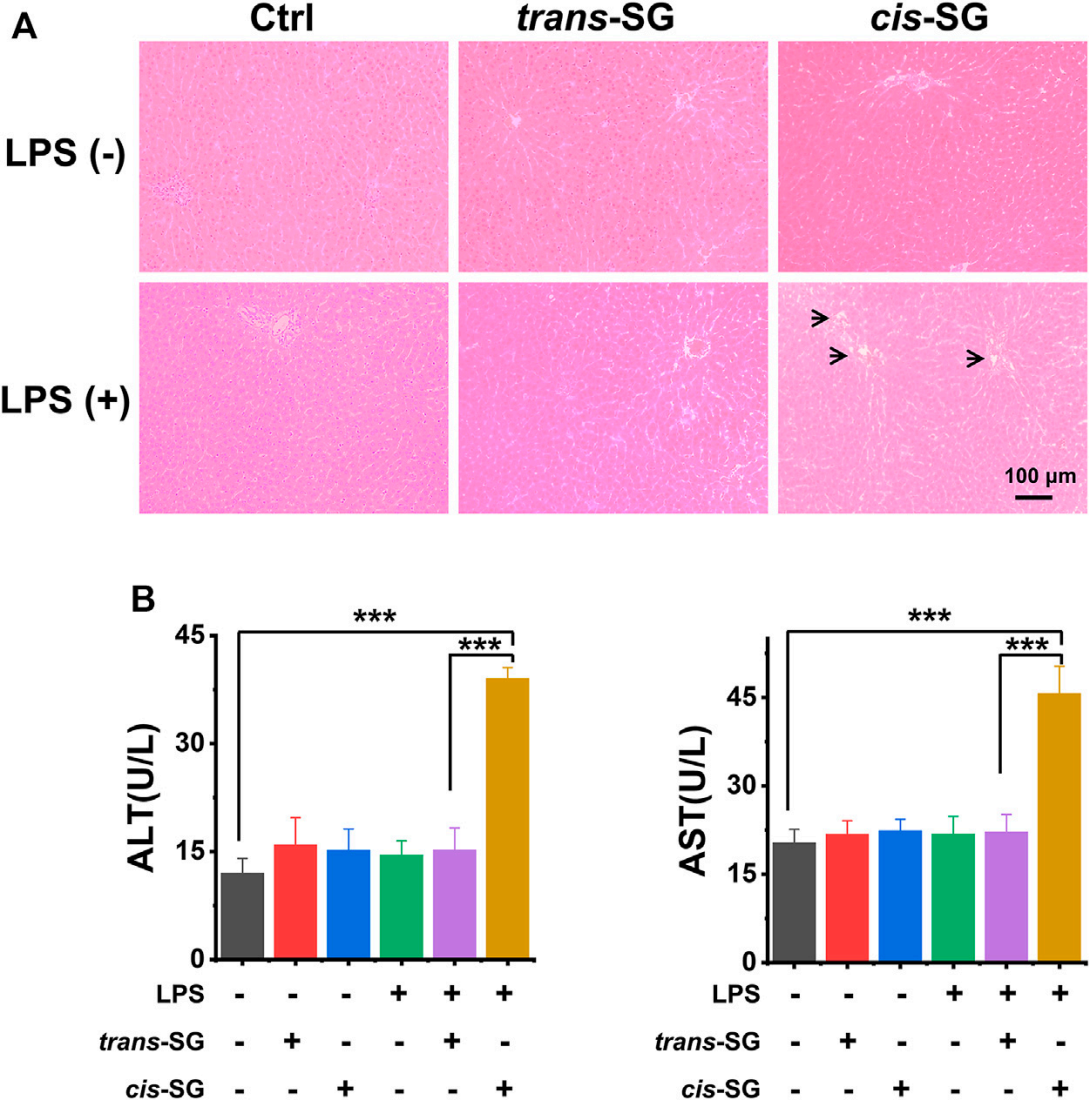
Hepatotoxicity evaluation of *trans-SG* and *cis-SG* in LPS-treated rat model. **(A)** The histological analysis of the liver sections with the treatment of *trans-SG* or *cis-SG* in the LPS-treated rat model (*n* = 9). **(B)** The serum ALT and AST levels. Scale bar = 100 μm. Values are expressed as mean ± SD. ***p < 0.001.

To determine which of the two isomers was associated with hepatotoxicity, we administered isolated *trans-SG* and *cis-SG* to LPS-treated rats to compare the hepatotoxic intensity of the two compounds. Results in Figure 2A show that the administration of *cis-SG* and LPS result in liver histologic damage and significantly increased plasma alanine aminotransferase (ALT) and aspartate aminotransferase (AST) (Figure 2B). By contrast, the administration of *trans-SG* and LPS was not associated with liver injury, suggesting that *cis-SG* is the major hepatotoxic component.

### The Higher Hepatotoxicity Induced by *Cis-SG* Could Be Detected in 3D Cultured Hepatic Cells

**FIGURE 3:**
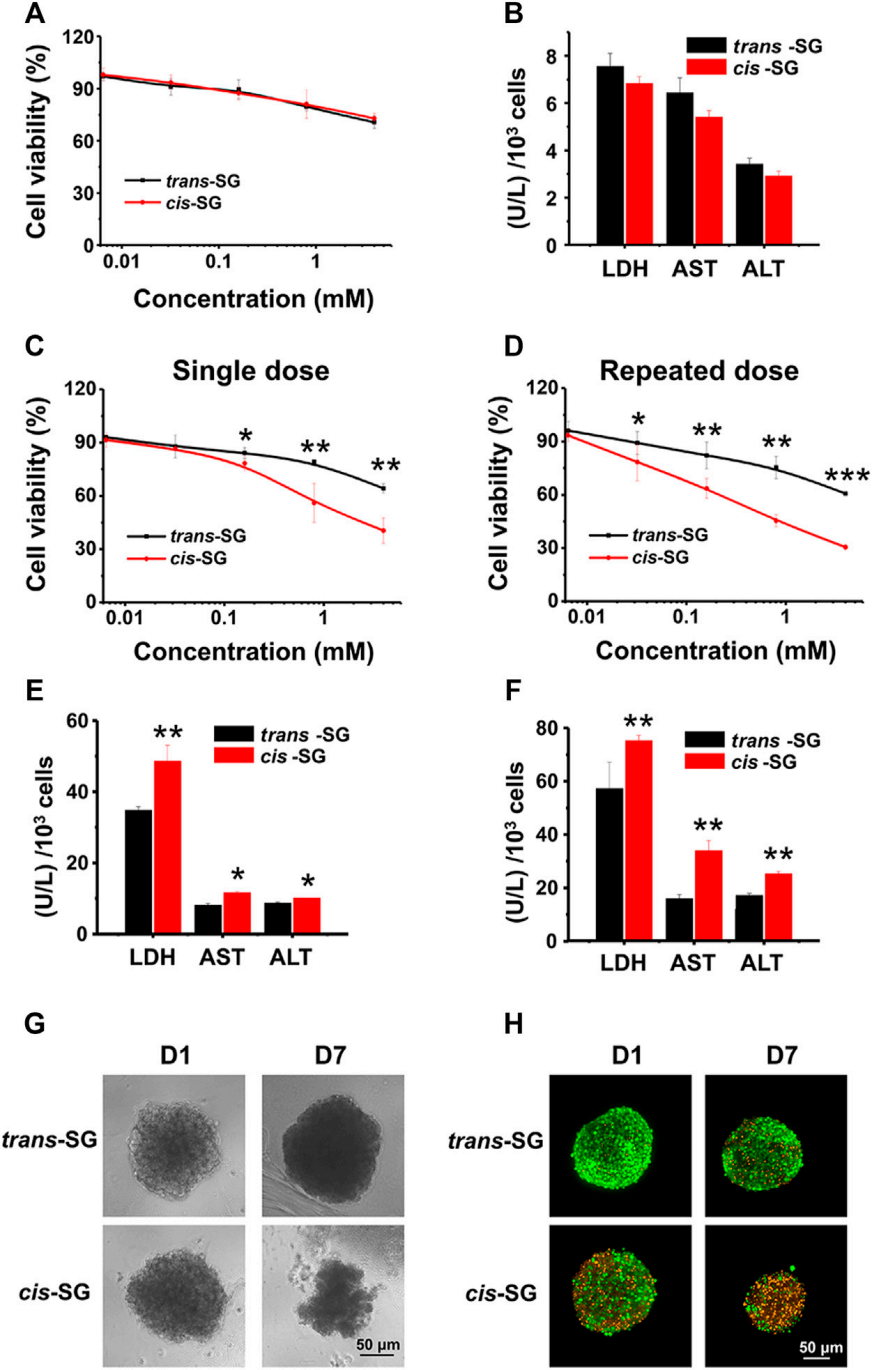
Organoid-based *in vitro* evaluation differentiated hepatotoxicity of *cis-SG* and *trans-SG* isomers. **(A,B)** Cell viability analysis **(A)** and biochemistry test (LDH, AST, and ALT) **(B)** on 2D cultured HepaRG cells showing no difference between the two isomers after 24 h of drug exposure. **(C,D)** Cell viability analysis of *trans-SG* and *cis-SG* on 3D cultured HepaRG organoids with single-dose **(C)** or repeated-dose **(D)** treatment. **(E,F)** The LDH, AST, and ALT levels of 3D cultured HepaRG organoids after being treated with *trans-SG* and *cis-SG* in single-dose **(E)** or repeated-dose **(F)**. **(G)** The morphological images of HepaRG organoids after being treated with *trans-SG* and *cis-SG* for 1 or 7 days. **(H)** The fluorescent images of HepaRG organoids after being treated with *trans-SG* and *cis-SG* for 1 or 7 days with live (green)/dead (orange) staining. Scale bar = 50 μm. Values are expressed as mean ± SD. *p < 0.05; **p < 0.01; ***p < 0.001.

To further reveal the possible hepatotoxic mechanism induced by *cis-SG*, the monolayer cultured HepaRG cells were used to detect the hepatotoxicity of *cis-SG* and *trans-SG*. However, there was no difference between *cis-SG* and *trans-SG*–induced hepatotoxicities. The cell viability and related indicators, such as lactate dehydrogenase (LDH), ALT, and AST, did not show any statistical difference with the treatment of *cis-SG* and *trans-SG* (Figures 3A,B).

Therefore, the model which could reflect the hepatotoxic difference between *cis-SG* and *trans-SG* was required. Next, the 3D cultured HepaRG organoids were further prepared to assess *cis-SG* and *trans-SG*–induced hepatotoxicities. Fortunately, due to the enhanced hepatic functions, the results obtained from the 3D cultured HepaRG organoids were consistent with earlier overall animal outcomes. By using the established 3D cultured organoids model, the significantly different hepatotoxic potential between the *cis-SG* and *trans-*SG stereoisomers was observed *via* either single or repeated dose. The *cis-SG* showed higher toxicity with the lower half-maximal inhibitory concentration (IC_50_) than did *trans-SG* in single- or repeated-dose treatment on cells (Figures 3C,D). In addition, the *cis-SG* and *trans-SG*–induced cell death was in a concentration-dependent manner. The activity of LDH, ALT, and AST was significantly increased in *cis-SG*–treated cells (Figures 3E,F). Similarly, the recorded morphological images also confirmed that *cis-SG* caused more serious cell damage with visible loss of cells and impairment of spheroid structure, especially with the repeated-dose treatment (Figure 3G). Live/dead fluorescence staining was also used to qualitatively indicate the viability of the organoids with drug treatment. Figure 3H showed that more cells were alive (green) in *trans-SG*–treated organoids than in *cis-SG*–treated organoids. These aforementioned results suggest that long-term medication may increase the risk of liver injury. More importantly, the 3D HepaRG organoid culture system can be used to reveal the possible mechanism of hepatotoxicity induced by *cis-SG*.

### Mitochondrial Damage Associated With *Cis-SG*–Induced Hepatotoxicity

**FIGURE 4:**
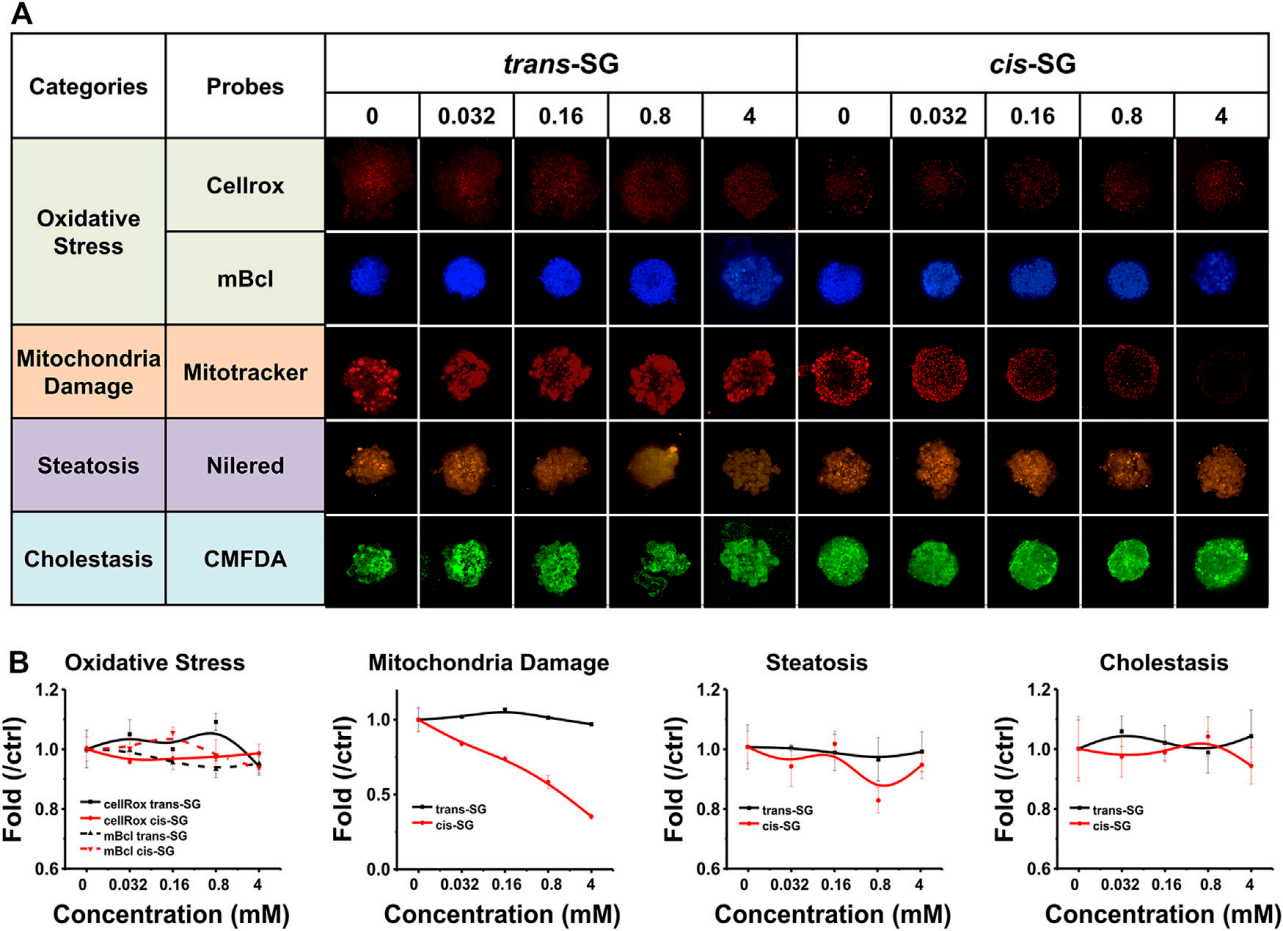
Multiparametric HCA of assessments on 3D cultured HepaRG organoids with *trans*-SG and *cis*-SG exposure for 24 h. **(A)** Representative confocal images shown corresponding to *trans-SG* and *cis-SG*. After incubation, 5 fluorescence-based endpoints were measured to assess chemically induced alterations in cellular function. **(B)** Image analysis readouts were derived to evaluate four kinds of different cellular alterations, including oxidative stress, mitochondria damage, steatosis, and cholestasis. Values are expressed as mean ± SD.

To demonstrate the potential mechanisms of drug related to hepatotoxicity, multiparametric analysis of assessments in 3D cultured HepaRG organoids treated with *trans-SG* or *cis-SG* have been carried out. The cells were stained with selected fluorescence probes and monitored by high content imaging and analysis (HCA) after being treated with *trans-SG* or *cis-SG* for 24 h. HCA allows observation of drug-induced hepatotoxic alterations using hyper-multicolor cellular imaging. Different molecular probes were used to indicate the majority of reported changes in oxidative stress, mitochondria damage, steatosis, and cholestasis. For oxidative stress, the generation of reactive oxygen species (ROS) and depletion of intracellular glutathione (GSH) were detected by CellROX™ Deep Red Reagent and monochlorobimane (mBCl). As shown in Figure 4A, both *trans-SG* and *cis-SG* did not cause oxidative stress in cells. For mitochondrial damage, the change in mitochondrial membrane potential is the main reason for mitochondrial dysfunctions. MitoTracker^®^ Deep Red FM was used to analyze the alteration of mitochondrial membrane potential. *cis-SG* could induce a significant decrease in fluorescence intensity, indicating the decrease of mitochondrial membrane potential (Figure 4B). For drug-induced steatosis, it indicates to the overaccumulation of lipids, mainly triglycerides, inside hepatocytes. Here, Nile red was used to stain and quantify the accumulation of neutral (e.g., triglycerides) and polar (e.g., phospholipids) lipids by its uptake and retention in cells. The images and quantified results showed that *trans-SG* and *cis-SG* did not cause steatosis. For cholestasis, the hepatic bile acid transporter substrate 5-chloromethylfluorescein diacetate (CMFDA) was used to stain the canalicular structures. Similar to the steatosis result, there was almost no alteration to the bile canaliculus structures of the 3D cultured HepaRG cells after being treated with *trans-SG* or *cis-SG*. These results demonstrated that the *cis-SG*–induced hepatotoxicity was mainly because of mitochondrial damages in the cells, and these results assist in gaining an understanding of the action mechanism of *P. multiflorum*–induced hepatotoxicity.

### Protecting Mitochondria Blocked *Cis-SG*–Induced Hepatotoxicity

**FIGURE 5:**
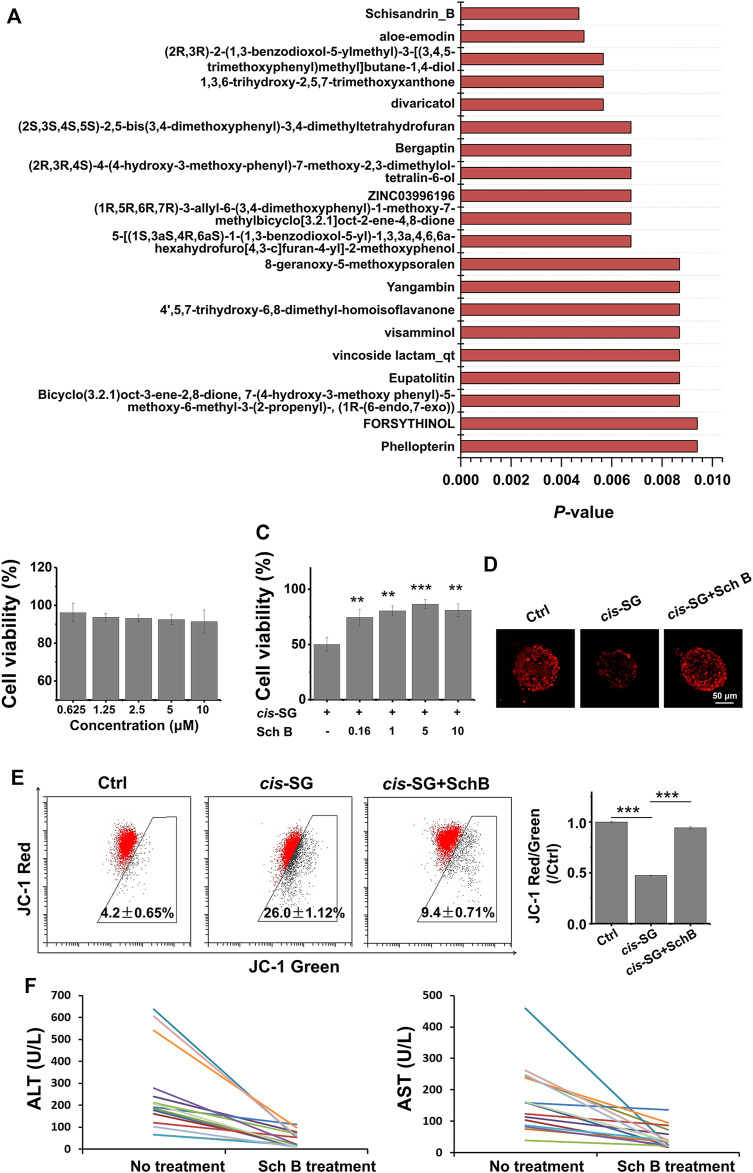
Protective effects of the computer-screened mitochondria protective agent and its preparations against liver injury on organoids and patients. **(A)** The Top 20 mitochondrial protective compounds screened by computer-aided network pharmacology approach from the natural products database. **(B)** The cell viability of HepaRG cells after being only treated with Sch B for 24 h. **(C)** The HepaRG cell viability recovered after being co-treated with Sch B and *cis-SG*. **(D)** The mitochondrial staining images of cells treated with different groups. **(E)** The flow cytometric results of HepaRG cells stained with the MitoProbe™ JC-1 Assay Kit, and the quantified ratio of JC-1 red fluorescence intensity to green fluorescence intensity, normalized to the control group. **(F)** Alleviation effect of the Chinese medicinal preparations containing Sch B on patients with *Polygonum multiflorum*–induced liver injury indicated by serum ALT and AST. Scale bar = 50 μm. Values are expressed as mean ± SD. **p < 0.01; ***p < 0.001.

Mitochondrial protective compounds were screened by a computer-aided network pharmacology approach from the natural products database which are listed in Table 2. Among these, Schisandrin B (Sch B) was ranked first and was available to be procured as a pure compound (Figure 5A). The herb containing Sch B, named *Schisandra chinensis*, is a well-used traditional Chinese medicine to treat acute liver injury in clinical applications. Hence, Sch B was selected as the mitochondrial protective agent to further verify the mechanism of *cis-SG*–induced liver injury. Firstly, the cytotoxicity of Sch B was tested. There was no significant hepatotoxicity to HepaRG organoids after being treated with Sch B in the concentration range of 0.625–10 μM (Figure 5B). When co-exposed with Sch B, the *cis-SG*–induced decrease of cell viability was significantly and dose-dependently ameliorated (Figure 5C). And by mitochondrial imaging, it was shown that the mitochondrial protective agent, Sch B, reversed the mitochondrial membrane potential, suggesting that Sch B could decrease mitochondrial damage induced by *cis-SG* (Figure 5D). The mitochondrial membrane potential was further detected by the MitoProbe™ JC-1 Assay Kit, which in the healthy mitochondria accumulates as a red fluorescent dimer and following mitochondrial injury is released into the cytoplasm as a green fluorescent monomer. As shown in Figure 5E, the flow cytometric results showed that the co-treatment of *cis-SG* and Sch B effectively reduced the *cis-SG*–induced mitochondrial damage. In addition, the hepatoprotective effect of the preparations containing Sch B was demonstrated by clinical data with the decrease of ALT and AST levels in patients’ serum (Figure 5F and Table 3).

**TABLE 2:** Network pharmacology predictions of mitochondria protective agents (Top 20) from natural products.

Rank	Predicted compounds	Matched total	p-value
1	Schisandrin B	6\15	0.00469
2	Aloe-emodin	7\20	0.00489
3	Divaricatol	5\11	0.00566
4	1,3,6-trihydroxy-2,5,7-trimethoxyxanthone	5\11	0.00566
5	(2R,3R)-2-(1,3-benzodioxol-5-ylmethyl)-3-[(3,4,5-trimethoxyphenyl)methyl]butane-1,4-diol	5\11	0.00566
6	5-[(1S,3aS,4R,6aS)-1-(1,3-benzodioxol-5-yl)-1,3,3a,4,6,6a-hexahydrofuro[4,3-c]furan-4-yl]-2-methoxyphenol	6\16	0.00676
7	(1R,5R,6R,7R)-3-allyl-6-(3,4-dimethoxyphenyl)-1-methoxy-7-methylbicyclo[3.2.1]oct-2-ene-4,8-dione	6\16	0.00676
8	ZINC03996196	6\16	0.00676
9	(2R,3R,4S)-4-(4-hydroxy-3-methoxy-phenyl)-7-methoxy-2,3-dimethylol-tetralin-6-ol	6\16	0.00676
10	Bergapten	6\16	0.00676
11	(2S,3S,4S,5S)-2,5-bis(3,4-dimethoxyphenyl)-3,4-dimethyltetrahydrofuran	6\16	0.00676
12	Bicyclo(3.2.1)oct-3-ene-2,8-dione	5\12	0.00869
13	Eupatolitin	5\12	0.00869
14	Vincoside lactam	5\12	0.00869
15	Visamminol	5\12	0.00869
16	4',5,7-Trihydroxy-6,8-dimethyl-homoisoflavanone	5\12	0.00869
17	Yangambin	5\12	0.00869
18	8-Geranoxy-5-methoxypsoralen	5\12	0.00869
19	Tetrahydrofuroguaiacin B	6\17	0.00939
20	Dauriporphinoline	6\17	0.00939

**TABLE 3:** The clinical data of DILI patients in this study.

No.	Age (years)	Gender	BMI	RUCAM score	Before intervention		After Sch B treatment	
					ALT (U/L)	AST (U/L)	ALT (U/L)	AST (U/L)
1	41	Female	22.81	7	177	160	10	21
2	15	Female	24.83	8	161	104	21	17
3	56	Female	22.43	8	185	241	19	73
4	22	Male	32.77	8	240	114	79	58
5	17	Male	20.41	7	637	460	54	25
6	38	Male	17.84	7	66	76	21	34
7	12	Female	18.96	6	191	159	113	136
8	53	Female	20.31	8	120	124	54	86
9	28	Male	23.72	7	211	39	69	22
10	25	Female	20.76	8	278	83	22	23
11	56	Female	23.73	9	65	86	18	38
12	39	Male	24.20	8	541	238	98	95
13	49	Female	35.94	7	102	247	9	20
14	19	Male	20.52	7	605	261	60	39
15	34	Female	20.31	7	207	162	11	36

**FIGURE 6:**
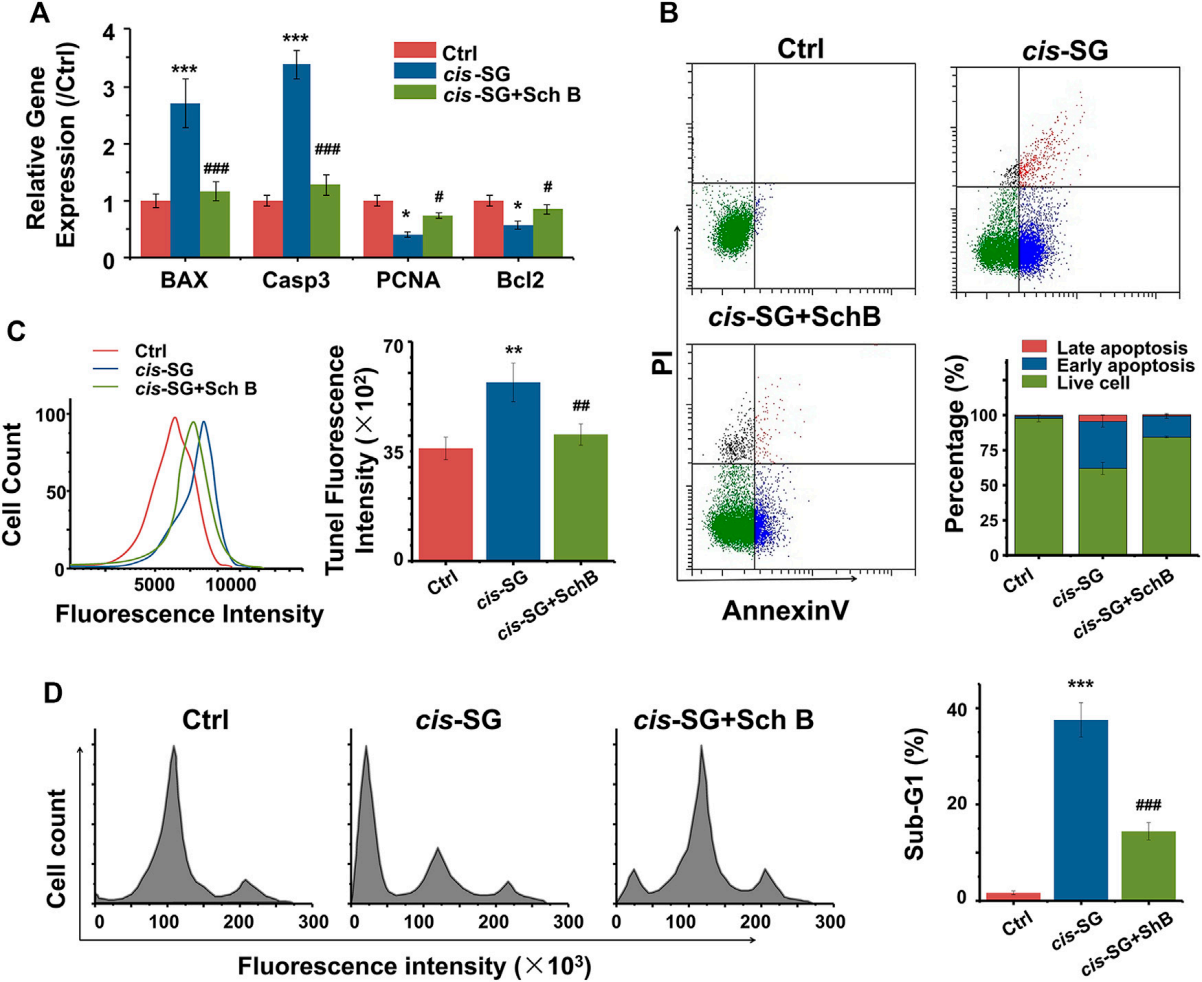
Inhibiting apoptosis was involved in the blocking effect of mitochondria protective agents against *cis-SG*–induced hepatotoxicity on 3D cultured HepaRG organoids. **(A)** Sch B significantly reversed the *cis-SG*–induced abnormal expressions of mitochondrial dysfunction–related genes (BAX, Casp3, PCNA, and BCL2). **(B)** Flow cytometry assays of Annexin V/PI staining showed Sch B significantly decreased the proportions of early and late apoptosis induced by *cis-SG*. **(C)** TUNEL analysis showed Sch B significantly decreased *cis-SG*–induced late apoptosis. **(D)** Cell cycle–based apoptosis assay for HepaRG organoids showed that Sch B significantly reversed the *cis-SG*–induced DNA fragmentation. Values are expressed as the mean ± SD. *p < 0.05, **p < 0.01, and ***p < 0.001, *cis-SG*–treated group *vs* control group; #*p <* 0.05, ##*p <* 0.01, and ###*p <* 0.001, *cis-SG* and Sch B co-treated group *vs cis-SG*–treated group.

Mitochondrial damage always induces cell apoptosis. *cis-SG*–induced apoptosis, indicated by either an increase of BAX and Caspase3 or a decrease of PCNA and Bcl2 gene expressions, was significantly reversed to control group levels (Figure 6A). Further analysis by flow cytometry showed that *cis-SG* induced 33.58% early apoptosis and 4.41% late apoptosis, while the combination of Sch B and *cis-SG* treatment showed a decreased proportion of either early or late apoptosis to 15.06% and 0.91%, respectively (Figure 6B). The inhibitory effect of Sch B on *cis-SG*–induced late apoptosis was also demonstrated by TUNEL analysis (Figure 6C). In addition, cell apoptosis was accompanied by DNA fragmentation. As shown in Figure 6D, the proportion of sub-G1 was increased after being treated with *cis-SG*, and Sch B could inhibit this effect.

## Discussion and Conclusion

It is obviously important for an accurate *in vitro* evaluation model to reflect the *in vivo* organ circumstances as closely as possible and thus can predict drug hepatotoxicity and identify the possible mechanisms (Teschke et al., 2015). Because 3D cultured organoids can simulate the intercellular contacts of organs and provide a suitable microenvironment to maintain the organ-level molecular cell phenotypes (Pampaloni et al., 2007; Ravi et al., 2015), they are more competitive than monolayer cultured cell models in evaluating hepatotoxicity (Lauschke et al., 2016; Andersson 2017). In our previous work, we developed a hepatic cell organoids culturing system with the liver-specific extracellular matrix and HepaRG cells (Wang et al., 2011; Juan et al., 2018). Regarding the liver-specific microenvironment, the functional differences between the 2D cultured cells and organ-level hepatocytes are dramatically great. Considering many hepatotoxic drugs that injure liver cells *via* their metabolites (especially metabolizing enzyme-activated metabolites), the 3D cultured hepatic organoids model is more suitable to predict drug hepatotoxicity. Notably, most of the clinical drug-induced liver injuries occurred in a delayed pattern (usually days or months of repeated medication), and the organ concentration of drugs was relatively low. However, the current *in vitro* studies usually used significantly high concentrations and single or limited exposure time in evaluation, which have limited references to understanding the clinical hepatotoxicity. The hepatic organoids model is suitable to test the repeated or accumulative intoxication of drugs at low concentrations due to its long-term culturing nature. This is so important that the test concentration of drug compounds would be comparable to *in vivo* organ concentration, and therefore, this will represent more accordant results of hepatotoxic potential.

For drugs, although they have the same functional groups but only a stereoscopic difference in structure, there might be divergent activity or toxicity of stereoisomers (Srinivas et al., 2001; Shim et al., 2016). The stereoscopic structural differences of drug candidates are sometimes ignored in drug research and development; for instance, there are 25% of marketed drugs of mixed isomers but not the single isomer (Hutt, 2002). However, the stereoisomers are difficult to differentiate regarding hepatotoxicity. Currently, there is not much concern about the toxicity of stilbene derivatives and their stereoisomers. The *trans-*form stilbenes, such as *trans-*resveratrol and *trans-SG*, are generally considered to be of good safety as potent drug candidates, as well as for their *cis-*isomers (Wang and Chatterjee, 2017). However, we have discovered that the *cis-SG*, other than the *trans-*form, contributed as the major hepatotoxic component in clinical cases and animal models (Li et al., 2017). The hepatotoxic potential of *cis-*stilbenes has not been addressed before, despite the isomerization of *trans-*stilbenes to their *cis-*forms occurring spontaneously when being exposed to ultraviolet light or sunlight (Koppen et al., 2012; Sharma et al., 2017; Xiao et al., 2018). Thus, there is a great need to elucidate the divergent hepatotoxicity and lesion mechanisms of structurally similar drug candidates. By using the established hepatic organoids model, we demonstrated the differential hepatotoxicity of the *cis-* and *trans-*isomers *in vitro* for the first time. By contrast, there was no difference in cytotoxicity between the two isomers in the common 2D cultured hepatocyte model. Interestingly, there was much more difference in hepatotoxicity when the culture time was prolonged for the two isomers. This suggested that there might be delayed or accumulated hepatotoxicity of the toxic isomer, which was in accordance with the clinical onset characteristics of DILI (Zhu et al., 2016). Combined with HCA, we further discovered the lesion mechanism of the hepatotoxic *cis-SG*, focusing on impairment of the mitochondria instead of the other common hepatotoxic mechanisms, such as oxidative stress, cholestasis, and steatosis. In order to further confirm the mitochondrial lesion mechanism, we screened and used the mitochondrial protective reagent to co-administer with *cis-SG*. The results showed that if we protected the mitochondria, the hepatotoxicity of *cis-SG* was partly blocked. This result demonstrated that the hepatotoxicity of *cis-SG* is mitochondria-dependent and illustrated the reliability of HCA results. Nevertheless, the more specific toxicity target of *cis-SG*–induced mitochondrial lesion needs to be further studied. These results suggested a necessary concern on the toxicity of *cis-*form stilbenes rather than their normally occurring *trans-*stereoisomers.

This study illustrated the utility of the organoids-based high-content imaging approach to differentiate and predict organ hepatotoxicity of a pair of *cis-* or *trans*-stereoisomers. The results also highlighted the remarkable influence of stereoisomerism on the toxicity of lead compounds in new drug development. Considering the liver as a complicated organ consisting of either parenchymal cells or non-parenchymal cells, it is interesting to test if there is an additional influence of non-parenchymal cells on the pathogenesis of drug hepatotoxicity in the organoids model. More specially, the resident and recruited immune cells in this organ might play pivotal roles in DILI pathogenesis (Kim et al., 2017), but this organ-level multiple cell typic interactions on hepatotoxicity in organoids are nearly unknown. A most recent study demonstrated that the transwell co-culture of hepatocytes with macrophages had the additional release of pro-inflammatory cytokines after drug exposure (Wewering et al., 2017). Thus, it is of great interest to establish the hepatocytes and immune cells co-cultured organoids and test drug hepatotoxicity in the future. Although there was no positive result of *cis-SG* causing injuries to cholangiocyte-like cells, the established organoids model has the ability to evaluate the potential of drugs to cause cholestasis due to the ability of HepaRG cells to differentiate into both hepatocytes and cholangiocytes in 3D cultured organoids. In summary, using the *in vitro* organoids model to simulate *in vivo* organ-level circumstances is important to evaluate and predict drug toxicity and thus decreases the possibility of drug research and developmental failure and withdrawal caused by unpredicted severe adverse drug reactions.

## Data Availability

The original contributions presented in the study are included in the article; further inquiries can be directed to the corresponding authors.
